# Examining the intertwined development of prosocial skills and ASD symptoms in adolescence

**DOI:** 10.1007/s00787-018-1114-3

**Published:** 2018-01-30

**Authors:** Anoek M. Oerlemans, Nanda N. J. Rommelse, Jan K. Buitelaar, Catharina A. Hartman

**Affiliations:** 1 Department of Psychiatry, Interdisciplinary Center Psychopathology and Emotion Regulation (ICPE), University of Groningen, University Medical Center Groningen, Groningen, The Netherlands; 2 Department of Cognitive Neuroscience, Donders Institute for Brain, Cognition and Behaviour, Radboud university medical center, Nijmegen, The Netherlands; 3 Karakter Child and Adolescent Psychiatry University Centre, Nijmegen, The Netherlands; 4 Department of Psychiatry, Donders Institute for Brain, Cognition and Behaviour, Radboud university medical center, Nijmegen, The Netherlands

**Keywords:** Autism spectrum disorders, Prosocial skills, Development, Adolescence

## Abstract

Autism spectrum disorder (ASD) and reduced prosocial behaviour are strongly intertwined. However, social interactions with peers may be increasingly practiced over the course of development and may instigate a reduction in ASD symptoms and vice versa. We, therefore, sought to determine if, during adolescence, possible improvements in prosocial behaviours and ASD symptoms may benefit one another over time. Participants were 2773 adolescents from the Tracking Adolescents’ Individual Lives Survey (TRAILS) cohorts. Measurements took place over three waves (mean ages: 11.1, 13.4, and 16.2 years). Longitudinal associations between teacher-rated classroom prosocial skills and parent-rated ASD symptoms were examined using the random intercept cross-lagged panel model (RI-CLPM). In addition to estimating the stable, between-person associations, the dynamical effects between prosocial skills and ASD symptoms over time were estimated at the within-person level. At the between-person level, prosocial skills and ASD symptoms were substantially negatively correlated. At the within-person level, a small and unexpected positive cross-lagged effect from wave 1 ASD symptoms on wave 2 prosocial skills was observed. We added to the existing literature by showing that, in addition to replicating the already firmly established between-person association between low prosocial skills and ASD, within-person gains in prosocial skills do not lead to subsequent reduction of ASD symptoms, and reductions in ASD symptoms do not lead to subsequent enhancement of prosocial skills. We, therefore, conclude from our findings that the inverse association between autistic symptoms and prosocial skills in adolescence is highly stable.

**Electronic supplementary material:**

The online version of this article (10.1007/s00787-018-1114-3) contains supplementary material, which is available to authorized users.

## Introduction

Autism spectrum disorders (ASD) are a group severely impairing neurodevelopmental disorders, characterized by impairments in social interaction and communication as well as restricted, stereotyped, and repetitive behaviour [1]. ASD symptoms typically manifest early in life (i.e., before age 3), but in particular less severe forms of ASD may be masked by learned strategies and may not become fully manifest until social demands exceed limited capacities [1]. ASD is highly heritable [with estimates ranging from 58 to 90%; 2–4] and in most cases stable over time. At the core of ASD are difficulties in perspective taking and, thereby, responding to others’ needs. This is also called an impaired ‘theory of mind’ [5]. As a consequence, spontaneous (i.e., without very explicit cueing) prosocial skills are usually greatly reduced in individuals with ASD [6, 7]. Prosocial skills refer to a number of behaviours intended to support others, including helping, sharing, comforting, co-operating, and volunteering [8]. These reduced prosocial skills impede the formation of reciprocal social relationships, as helping, sharing, comforting, co-operating, and volunteering are defining features of friendships.

As with all skills, gains in prosocial behaviour are made when these skills are practiced through frequent interaction with parents, siblings, and peers. It is through observing, modelling, practicing, and receiving positive feedback that an individual acquires prosocial knowledge, skills, and behaviour that become more automated and an inherent part of one’s behaviour patterns and personality [9]. Social learning starts early in life. In typical development, infants are already being trained in social behaviour, such as in the interchange of gazing and looking away and of cooing and responding through early face-to-face interactions with caregivers. Through imitation, young children communicate social interest in others and initiate interaction with others [10]. Social learning is also under continuous development; the elementary social skills of the infant enable him to acquire the more advanced ones of the toddler, pre-schooler, teenager, and so on [9]. Progress in social learning is rewarded, because social competence is beneficial and participating in social exchanges in itself is experienced as enjoyable. This is an important reinforcement for repeating and consequently preserving social acts. Continuous development is important, since the prosocial skills which serve a 5-year-old child are clearly not adequate for negotiating the more complex social world of a teenager. For instance, handing over a toy for comfort may be adequate for a 5-year-old, but not for a teenager. Prosocial skills heavily rely upon the development of other competencies, including cognitive abilities and emotion regulation skills [11]. A child must have the cognitive ability to notice and interpret social cues, the emotional capacity to empathize with the other person, recognize that someone needs help, and subsequently decide to help or not by selecting and performing an appropriate behaviour for that situation. In addition, the child must have knowledge of social rules, memory of past experiences, and expectations for future experiences [11, 12].

In children with ASD, the social learning cycle is disrupted. From an early age, children who later developed autism show less interest in back-and-forth sharing of sounds, smiles, or other facial expressions, and exhibit significant deficits in imitation skills [10]. Their cognitive development is also delayed, with difficulty perceiving and understanding emotions and impaired theory of mind, resulting in further impairments in socialization and communication. Acquisition of new social skills may require greater effort than for the typical developing child, possibly reducing intrinsic interest in the social environment. As a result, the social learning cycle is increasingly lagging behind in many children with ASD, after which point they are no longer able to keep up with the increasingly complex and often implicit social rules. It has been shown that ASD is strongly related to fewer number of —and less intimate—friendships [8, 13]. Consequently, the opportunity of children with ASD to practice prosocial behaviours is increasingly reduced, resulting in increasing deficits.

One of the most relevant time windows for examining the association between prosocial behaviours and ASD symptoms is adolescence. Adolescence is a period of major developmental changes marked by physical, cognitive, and emotional growth. There is substantial variability in ASD-related symptoms and behaviours over time in adolescents. Although most individuals with ASD rarely move off the spectrum fully and need a lifetime of specialist support [14], several recent longitudinal studies confirm the existence of different developmental ASD trajectories in early and middle childhood [e.g., 15, 16]. Particularly, during adolescence, there is a tendency of modest improvement in clinical samples and symptom decline across studies [see for a review, 14]. Not withstanding, a substantial subset of youths with ASD show worsening of social withdrawal, irritability, and hyperactivity [17]. Adolescence should be considered a critical period for the development of prosocial behaviours. In adolescence, peer networks expand, close friendships become more and more important, and romantic relationships start to emerge [18]. These more complex social relationships provide a context in which adolescents learn more complex social skills (e.g., learning to respect the opinion of others, to include others in the decision making, and how to join a group) that are crucial for daily functioning [19]. The school context is important herein. Children spent most of their day at school socially interacting with peers, which gives them opportunity to practice their social skills. It is also the context in which successful social relationships can have added benefit to both social and academic development [20]. As such, although few studies have addressed this, understanding how prosocial skills and ASD symptoms relate to one another over the course of adolescence, particularly whether gains in prosocial skills may instigate a reduction of ASD symptom severity is of importance. It could tell us if improving prosocial skills (e.g., by training these as well as by increasing the opportunity to practice learned prosocial behaviours by helping individuals with ASD broaden their social network) may be a possible intervention strategy to improve outcome, not only in early childhood as is often done, but also in adolescence.

The current study set out to examine the longitudinal, reciprocal association between prosocial skills and ASD symptoms. Continuous parent-reported ASD symptom questionnaire data and teacher ratings of prosocial behaviour in the classroom were available for a large population-based and clinical-referred sample of pre-adolescents who were followed up three times until late adolescence in The Netherlands (the combined TRAILS population and clinical-referred cohort). The proportion of adolescents with (sub)clinical ASD symptom levels was much higher in a sample of adolescents who sought psychiatric help at any time in their life, than in a purely population-based sample. Combining the two cohorts thus allowed us to examine the association between prosocial skills and ASD symptoms across the full spectrum of symptom severity. We aimed to examine whether prosocial skills predicted ASD symptoms at a subsequent timepoint above and beyond the effect of ASD symptoms, and vice versa, whether ASD symptoms predicted prosocial skills at a subsequent timepoint, above and beyond the effect or prosocial skills. Prosocial skills were teacher-rated, representing real life prosocial behaviours towards peers in a classroom setting, an important developmental context in adolescence. Given that, during adolescence, there tends to be some ASD symptom reduction on one hand and an increased interest in peers and consequent practicing of social skills on the other hand, we expected to find bidirectional negative within-person effects over time from prosocial behaviour to ASD symptoms, and from ASD symptoms to prosocial skills (i.e., adolescents with fewer ASD symptoms at wave 1 would be expected to have higher scores on prosocial skills at wave 2 and similarly, adolescents who have better prosocial skills at wave 1 are expected to have fewer ASD symptoms at wave 2). This in addition to a between-waves stable and negative association between ASD symptoms and prosocial skills.

## Method

### Participants and procedure

**Fig. 1:**
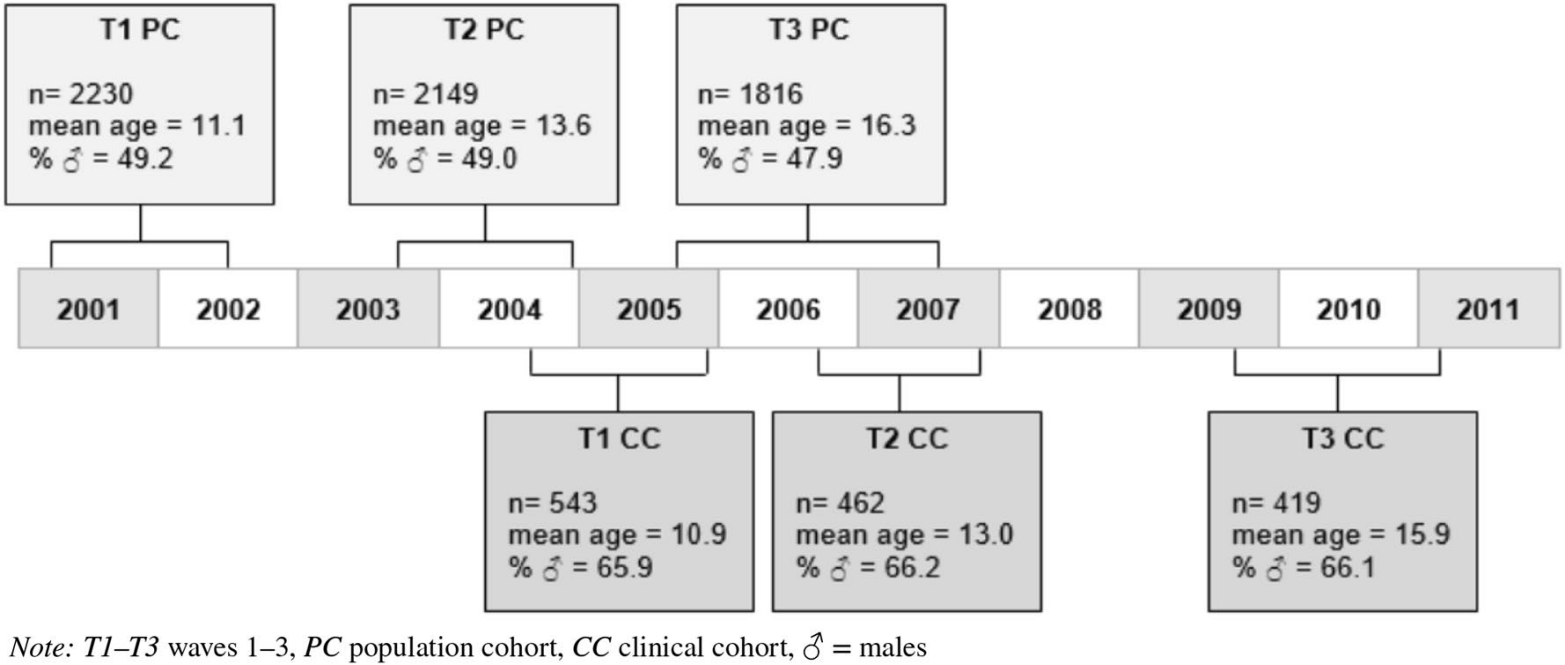
TRAILS study design

The present study uses data from the first three waves of the TRacking Adolescents’ Individual Lives Survey (TRAILS) and the clinical-referred cohort of TRAILS (TRAILS-cc). TRAILS is a prospective cohort study of Dutch adolescents, with bi- or triennial follow-up assessments, see Fig. 1. The TRAILS target sample comprised of young adolescents from five municipalities in the north of The Netherlands, including both urban and rural areas. Participants for TRAILS-cc were recruited from a large child psychiatric outpatient clinic in the Northern Netherlands with the same target area as covered by the population sample. Children aged 10 through 12 years who had been referred to this outpatient clinic at any point in their life and regarding any type of mental health problem were eligible for participation in TRAILS-cc. The research protocols of the TRAILS population and clinical-referred cohorts are identical for maximal comparability, i.e., the latter cohort was set up with the aim to increase variability in psychopathology by oversampling children with problems. The current report is based on data from the first three assessment waves (*T*1–*T*3).

At baseline, *N* = 2230 children from a population cohort and *N* = 543 children from a clinical cohort agreed to participate (total *N* = 2773). Two children had missing data on all dependent variables studied here and were excluded. Although TRAILS-cc was not an ASD-only sample, about 40% of the participants (*n* = 214) had a clinical DSM-IV-based diagnosis of ASD based on data linking with the Psychiatric Case Register-North Netherlands (PCR-NN). The PCR-NN registers specialist mental healthcare (MHC) use of Dutch inhabitants of the Northern regions of The Netherlands since 2000 [described in more detail 21]. An additional 33 participants from the TRAILS population cohort (as listed by the PCR-NN) had a clinical diagnosis of ASD, making the total number of individuals with a lifetime ASD diagnoses 247, see Table 1 for sample characteristics. The number of individuals with a lifetime clinical ASD diagnoses (approximately 8.9% of the total sample; a rough estimate since, as described in the paper, the PCR-NN is not flawless [21], nor are all children with ASD referred) suggests that it is likely that our sample represented the full spectrum of ASD symptom severity (ranging from low-severe, non-clinical to highly-severe, clinically significant symptoms). A detailed description of the study design, sampling procedures, data collection, and measures of the TRAILS study is published elsewhere [22, 23]. The Dutch Central Committee on Research Involving Human Subjects approved all the TRAILS study protocols. All children and their parents provided written informed consent to participate. Response rates and descriptive statistics for the three waves are provided in Table 1.

**Table 1:** Sample characteristics

	Population cohort (PC)	Clinical cohort (CC)	Total sample
Baseline (B)			
*N*	2935	1264	4199
*T*1			
*N* (% RR)	2230 (76.0)	543 (43.0)	2773
Mean age in years (sd)	11.09 (0.55)	10.89 (0.61)	
% males	49.2	65.9	
IQ	97.19 (14.97) (range 45–149)	96.72 (15.52) (range 58–142)	
CSBQ			
% RR	97.4	99.1	97.7
*M* (sd)	5.77 (5.96)	14.10 (10.06)	
Prosocial behaviour^b^			
% RR^c^	86.5	89.9	87.1
*M* (sd)	29.30 (6.18)	25.69 (6.51)	
*T*2			
*N* (%RR)	2149 (96.4)	462 (85.1)	2611
Mean age in years (sd)	13.56 (.53)	12.96 (.62)	
% males	49.0	66.2	
CSBQ			
% RR	88.5	97.8	90.1
*M* (sd)	5.36 (6.08)	14.40 (10.35)	
Prosocial behaviour^b^			
% RR^d^	64.6	84.2	68.1
*M* (sd)	27.80 (5.93)	25.60 (6.71)	
*T*3			
*N* (%RR)	1816 (81.6)	419 (77.2)	2235
Mean age in years (sd)	16.30 (0.73)	15.91 (0.66)	
% males	47.9	66.1	
CSBQ			
% RR	82.5	97.9	85.4
*M* (sd)	5.26 (5.97)	14.04 (10.27)	
Prosocial behaviour^b^			
% RR^e^	49.1	78.5	54.6
*M* (sd)	28.12 (6.27)	23.64 (6.86)	
#Lifetime clinical ASD diagnoses	33	214	247

*N* number of participants, *B* baseline, *T1* wave 1, *T2* wave 2, *T3* wave 3, *% RR* response rate in percentages, *M* mean, *sd* standard deviation, *PC* population cohort, *CC* clinical cohort, *ns* non-significant

^a^IQ was derived from the Vocabulary and Block Design subtests of the Revised Wechsler Intelligence scale for Children (WISC-R) (administered at wave 1)

^b^The mean and sd of prosocial behaviour is based on the aggregated score of the nine selected items

^c^Participants with complete vs. incomplete *T*1 prosocial behaviour data did not differ significantly on mean CSBQ scores (PC: *M* = 5.82 vs 5.50, respectively, *p* = 0.404 and CC: *M* = 14.12 vs. 13.98, *p* = 0.924)

^d^Participants with complete vs. incomplete *T*2 prosocial behaviour data did not differ significantly on mean CSBQ scores (PC: *M* = 5.37 vs 5.34, respectively, *p* = 0.921 and CC: *M* = 14.57 vs 13.44, *p* = 0.402)

^e^Participants with complete vs. incomplete *T*3 prosocial behaviour data did not differ significantly on mean CSBQ scores (PC: *M* = 5.28 vs 5.24, respectively, *p* = 0.897 and CC: *M* = 14.50 vs. 12.32, *p* = 0.079)

### Measures

#### ASD symptoms

Parent-reported ASD symptoms were measured using the Child Social Behaviour Questionnaire (CSBQ) [24, 25]. The CSBQ contains 49 items that were rated on a three-point scale ranging from ‘does not apply or occur’ to ‘clearly or often applies. Items refer directly to DSM-IV criteria for autistic disorder, but also represent less severe variations of these criteria as well as ASD-associated problem such as executive function problems and disruptive behaviour in social settings [26]. The items aggregate into six subscales: ‘reduced contact and social interests’, ‘difficulties in understanding social information’, ‘stereotyped behaviour’ ‘fear of and resistance to changes’, ‘not tuned’, and ‘orientation problems’. Because the latter two subscales (i.e., ‘orientation problems’ and ‘not tuned’) are not specific for ASD (i.e., similar behaviours are scored in ADHD) and it was our aim to specifically focus on ASD symptoms, an aggregated measure (i.e., ASD core score) based on the first four subscales was used in this study [26, 27]. Multiple studies have shown that the CSBQ has good psychometric properties with regard to test–retest and interrater reliability, internal consistence of the scales (all reliability indices > 0.75), and good criterion validity for both for high-functioning children and for children with mild-to-moderate mental retardation [24, 27–30]. Overall internal reliability (Cronbach’s alpha: *T*1 = 0.89, *T*2 = 0.92, *T*3 = 0.92) was excellent.

#### Prosocial skills

Prosocial skills towards peers in the classroom setting were measured using a teacher questionnaire, developed for TRAILS. The questionnaire contained 11 items that were rated on a five-point scale ranging from ‘never’ to ‘always’ that captured spontaneous forms of prosocial skills, such as ‘Shows sympathy for someone who has made a mistake’, ‘Takes the interests of other children into account’, and ‘Apologizes when something goes wrong’ [31]. Seven of the eleven items were adapted from an earlier questionnaire on prosocial skills [32] and these were supplemented with four items derived from a study on solidarity [33]. We used the aggregated sum scores for the three timepoints in our analyses. Upon critical evaluation of the 11 items, two of them did not appear to directly assess prosocial skills towards peers, but rather assessed task-oriented behaviour (i.e., ‘student does not adhere to commitments’ and ‘student fails to finish tasks’). Therefore, we decided to base the aggregate sum score on the remaining nine items, see Table 2. Internal consistency was excellent (Cronbach’s alpha: *T*1: *α* = 0.92, *T*2: *α* = 0.92, and *T*3: *α* = 0.92).

**Table 2:** Overview of the items of the teacher-rated prosocial questionnaire

**1**	**Tries to stop a fight or argument**
**2**	**Invites bystanders to participate in a game**
3	Does not adhere to commitments
**4**	**Helps others to pick up objects that were dropped**
**5**	**Praises the work of other (less competent) students**
**6**	**Shows sympathy for someone who has made a mistake**
**7**	**Offers help to children struggling with a difficult task**
8	Fails to finish tasks
**9**	**Comforts upset children**
**10**	**Takes the interests of other children into account**
**11**	**Apologizes when something goes wrong**

Items that were included in the aggregated sum score are printed in **bold**. Items 3 and 8 were omitted from the sum score on theoretical grounds (i.e., they did not appear to directly assess prosocial behaviour directed to others, but rather assess task-oriented behaviour)

#### Intelligence

Intelligence was derived from the Vocabulary and Block Design subtests of the Revised Wechsler Intelligence scale for Children (WISC-R) (administered at wave 1) [34].

#### Social well-being among classmates

Adolescents’ self-reported social well-being among classmates (which includes liking, affection, and status) were assessed with a scale based on the Social Productions Function (SPF) Theory [35]. Answer categories ranged from 1 (never) to 5 (always). Higher scores indicated more positive feelings of well-being among classmates (therefore, less feelings of rejection in school). Internal consistency was good (Cronbach’s alpha: *T*1; *α* = 0.90; *T*2: *α* = 0.86, *T*3: *α* = 0.83).

### Data analysis

Descriptive statistics were calculated using SPSS version 20. Response rates and descriptive statistics for the three waves are provided in Table 1. For 80 participants, no prosocial data were collected in any of the three waves and these participants were excluded from further analyses. Excluded participants did not differ from the rest of the participants on sex (*p* = 0.112) and ASD symptom level (*p* values for all three assessment waves > 0.10). They were slightly older at each assessment than included participants [*T*1: *M* = 11.3 (0.7) versus *M* = 11.1 (0.5); *T*2 14.0 (0.5) compared to m = 13.4 (0.6); *T*3 16.7 (0.8) versus 16.2 (0.7), all *p* values < 0.001] and had a lower frequency of lifetime clinical ASD diagnosis (2.5 versus 9.1%, *p* = 0.041) as derived from the PCR-NN. Cases with incomplete data (i.e., missing data on 2 or less waves) were included in the analyses as Mplus provides a method for handling incomplete data using Full Information Maximum Likelihood Estimation (FIML) [36, 37].

First, we calculated correlations and intra-class correlations between the three prosocial skills and ASD symptom scores, respectively. Second, the longitudinal association between prosocial skills and ASD symptoms was examined by means of a random-intercepts cross-lagged panel model (RI-CLPM) (in Mplus version 6.11). In the RI-CLMP, variance at the within-level is distinguished from variance at the between-level and, therefore, constitutes a multilevel approach taking into account that measurements are nested within individuals. An important advantage of the RI-CLPM over the common CLPM is that it controls for time-invariant trait-like individuals differences (i.e., between-person effects) in prosocial skills and ASD symptoms, such that more insight is provided in how these two constructs are linked at an intra-individual (i.e., state-like) level [38]. Doing so is important, because applying findings from the aggregate (between-person) level to interpret causes and effects on the individual (within-person) level may result in an error of inference or ecological fallacy [38–40]. The RI-CLPM splits the observed score variance in two major parts. The first part contains the variance that is due to the individual’s stable position in the sample at the between-person level. This trait-like stability is captured with two random intercepts that were included in the model (one for prosocial skills and the other for ASD symptoms). The observed scores were indicators of these factors, and all factor loadings were constrained at 1. The second part contains the within-person fluctuations around a person’s own expected score. Expected scores are computed based on the rank-order position of an individual (as indexed by their person-mean level across the three waves) on the random intercept and the observed mean level structure over time. This results in an expected score for every individual for each measurement. These individuals’ expected scores follow the same pattern of change as the sample mean, but on a person-mean-adjusted level. However, above and beyond their expected score, there is unexplained variance, due to individuals’ temporal deviations from their own expected scores. In the RI-CLPM, this variance is captured by latent factors (one per measurement occasion, each loading constrained at one). Finally, the error variances of the observed score were constrained to be zero; therefore, all variation in the observed scores was completely captured by the within-person and between-person latent factor structure [39]. As a result, the interpretation of the cross-lagged effects derived from the RI-CLPM is different from those estimated in the common CLPM. The correlation between the random intercepts reflects how stable between-person differences in prosocial skills are linked with stable between-person differences in ASD symptom. The auto-regressive paths reflect to what extent within-person deviations in prosocial skills and ASD symptoms can be predicted by deviations from their own expected scores on prosocial skills and ASD symptoms, respectively (i.e., carry-over effects). The cross-lagged effects reflect whether prosocial skills and ASD symptoms are linked reciprocally, and indicate whether a deviation from their own expected score in prosocial skills predicts a deviation from their own expected score in ASD symptoms one measurement wave later (and vice versa). The within-person correlation at wave 1 reflects the extent to which a person’s individual deviation from their own expected score on prosocial skills at wave 1 is associated with the deviation from their own expected score on ASD symptoms at wave 1. The correlated residuals at waves 2 and 3 reflect the correlated change (i.e., the extent to which a within-person change in prosocial skills is associated with a within-person change in ASD symptoms, independently of the prosocial skills and ASD symptoms that were present at the previous wave) [39, 41]. We tested a reciprocal model that included auto-regressive paths of prosocial skills and ASD symptoms, respectively (i.e., from waves 1 to wave 2 and from waves 2 to 3), concurrent correlations, and bidirectional cross-lagged paths from prosocial skills to ASD symptoms at a later timepoint and vice versa. To evaluate the goodness of fit of the model, the following indices were included: (a) Chi square (*χ*^2^); (b) the Comparative Fit Index (CFI) and the Tucker–Lewis Fit Index (TLI) with good fit indicated by values > 0.95; (c) the Root Mean Square Error of Approximation (RMSEA) with values < 0.05 indicating good fit, and the Standardized Root Mean Squared Residual (SRMR) with values < 0.08 indicating good fit [42, 43]. The Satorra–Bentler-scaled Chi-square difference test was utilized to compare model fits [44]. We used Robust Maximum Likelihood Estimation to take into account the non-normal distribution of ASD symptom data [45].

To test invariance of the associations over time, we compared unconstrained models against models in which auto-regressive paths and cross-lagged coefficients were constrained to be equal over time. If two models fitted the data equally well, the most parsimonious was chosen (that is, if by adding equality constraints model fit did not deteriorate, the model with equality constraints was selected) [43]. If adding equality constraints on, for example, the cross-lagged paths deteriorates model fit, this means that the effects from wave 1 to wave 2 are not equal to the effects from wave 2 to wave 3 and thus—depending on the child’s age—the within-person cross-lagged associations between prosocial skills and ASD symptoms may be different. If model fit does not deteriorate by adding constraints, this means that the effects are similar for each time interval and not age-dependent.

Furthermore, we tested whether this final model fitted equally well across gender (male, female), cohort (population, clinically referred), diagnostic status (ASD cases versus non-cases), IQ (low versus high), and self-reported social well-being among classmates (low versus high based on median split across the three measurements waves). In addition, we reran the final model separately for ASD-social and communication behaviours (based on CSBQ subscales ‘reduced contact and social interests’ and ‘difficulties in understanding social information’) and for ASD-repetitive behaviours (based on CSBQ subscales ‘stereotyped behaviour’ and ‘fear of and resistance to changes’) to test whether the association between prosocial skills in the classroom and ASD depended on type of ASD symptoms.

Finally, because we oversampled children with (sub)clinical ASD symptom levels, our sample is not an accurate representation of the general population. To determine if oversampling had an effect on the findings, we conducted a sensitivity analysis, where we weighted the clinically referred cases based on the percentage of the general population they would have been (based on [46]; 4.2%). In our study, individuals who had been referred to and used youth mental health care [i.e., participants from the clinical-referred cohort + 4.2% (*n* = 89) individuals from the population cohort] make up 22.8% of our total sample. Based on these numbers we calculated a weighting factor (0.18 for those who had received care, 1.24 for the remaining of the population sample). We reran the optimal RI-CLPM model including the weighting factor.

## Results

### Correlations and intra-class correlations

ASD ratings were highly correlated (*T*1–*T*2: *r* = 0.77, *T*2–*T*3: *r* = 0.71, *T*1–*T*3: *r* = 0.77, all *p* values < 0.001). Prosocial skills were moderately correlated (*T*1–*T*2: *r* = 0.40, *T*2–*T*3: *r* = 0.32, *T*1–*T*3: *r* = 0.30, all *p* values < 0.001). Based on intra-class correlations, 75.5% of the variance in parent-rated ASD symptoms was explained by differences between persons (or stable traits), the remainder by within-person fluctuations. For teacher-rated prosocial skills, 34.2% of the variance was explained by differences between persons, the remainder by fluctuations within persons.

### Model comparisons

**Fig. 2:**
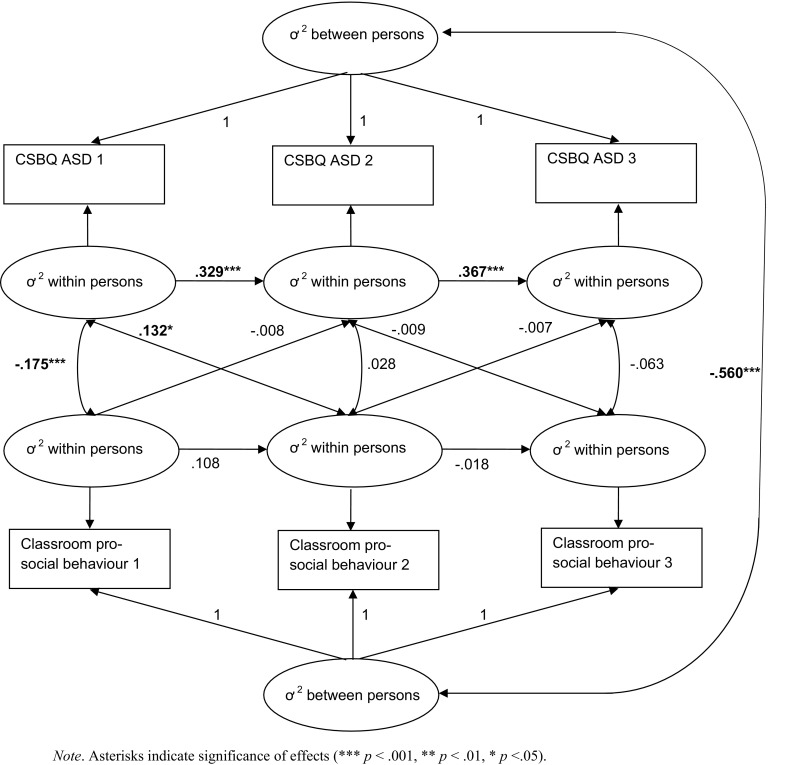
Optimal Random-Intercepts Cross-Lagged Panel Model (RI-CLPM) of the association between ASD symptoms and prosocial behaviour over time in the full sample. Asterisks indicate significance of effects (*** *p* < 0.001, ** *p* < 0.01, * *p* < 0.05)

The full model achieved good model fit [χ^2^ (1) = 2.80, *p* = 0.094, CFI = 0.999, TLI = 0.992, RSMEA = 0.026 (90% CI = 0.000–0.063), and SMRS = 0.009], see Table 3. Next, we added equality constraints over time to test whether it was possible to fix the auto-regressive paths between adjacent waves. This would tell us if carry-over effects were equal from wave 1 to wave 2 and from wave 2 to wave 3. No loss of fit was incurred by constraining the two auto-regressive paths for ASD symptoms [∆*χ*^2^ (1) = 0.012, *p* = 0.734], indicating that these path coefficients over time were equal. Fixing the auto-regressive paths for prosocial behaviour over time significantly impaired model fit [∆χ^2^ (1) = 6.02, *p* = 0.014]. Subsequently, we tested if the cross-lagged paths from ASD symptoms to prosocial behaviour were equal from waves 1 to 2 and from waves 2 to 3 by adding equality constraints over time. Fixing the cross-lagged paths significantly impaired model fit as evidenced by a significant Chi square change when the cross-lagged paths were constrained [∆χ^2^ (1) = 7.01, *p* = 0.008]. Fixing the cross-lagged paths from prosocial behaviour to ASD symptoms did not incur loss of fit [∆χ^2^(1) = 2.08, *p* = 0.149]. Thus, the most optimal model was the model with constrained auto-regressive ASD paths, unconstrained auto-regressive prosocial skills paths, unconstrained cross-lagged paths from ASD symptoms to prosocial skills, and constrained cross-lagged paths from prosocial skills to ASD symptoms, [χ^2^ (3) = 3.48, *p* = 0.323, CFI = 1.00, TLI = 0.999, RSMEA = 0.008 (90% CI = 0.000–0.034), and SMRS = 0.013], see Fig. 2 for parameter estimates derived from the selected model. At the between-person level, prosocial skills and ASD symptoms were substantially negatively correlated with each other (*ß* = − 0.560, *p* < 0.001), indicating that adolescents for whom higher levels of ASD symptoms were reported by parents across the three measurement waves had poorer prosocial skills according to teachers across the three waves. At the within-person level, a smaller positive cross-lagged effect from *T*1 ASD symptoms on *T*2 prosocial skills was observed (*ß* = 0.132, *p* = 0.029), indicating that within-person deviations in ASD symptoms at age 11 were predictive of within-person deviations in prosocial skills at age 13. When an adolescents’ score on ASD symptoms was higher than expected at age 11 (based on his/her person-mean-level across the three waves), the adolescents were reported to have better than expected prosocial skills 2 years later at age 13 (and vice versa). The observed positive within-person auto-regressive ASD paths (*T*1–*T*2: *ß* = 0.329, *p* < 0.001 and *T*2–*T*3: *ß* = 0.367, *p* < 0.001) indicate that within-person deviations in the level of ASD symptom can be predicted by the individual’s prior deviation from their own scores (e.g., when an individual has higher ASD symptoms than the expected mean at time 1, the individual also has higher ASD symptoms at the subsequent wave). For prosocial skills, no associations were found at the within-person level; auto-regressive paths as well as cross-lagged paths were all non-significant. The negative within-person correlation at the first wave (*ß* = − 0.175, *p* < 0.001) indicates that within-person deviations in ASD symptoms were negatively linked to within-person deviations in prosocial skills, in addition to between-person correlations. When an adolescents’ score on ASD symptoms was higher than expected at age 11, then his/her score on prosocial skills at age 11 was lower than expected (and vice versa).

**Table 3:** Fit statistics for the random-intercepts cross-lagged panel model (RI-CLPM)

	Fit statistics								Model comparisons (compared to the full model)	
	χ^2^	*df*	*p*	CFI	TLI	RSMEA	90% CI	SMRS	∆ χ^2^(*df*)^a^	∆ *p*
Step 0: Fit statistics for the full reciprocal model										
i. Reciprocal model with stability paths, within-wave correlations and bidirectional cross-lagged paths	2.80	1	0.094	0.999	0.992	0.026	0.000–0.063	0.009		
Step 1: Testing invariance of full model										
1. Both contemporaneous and cross-lagged paths constrained to be equal (= no change over time)	12.96	5	0.024	0.998	0.993	0.024	0.008–0.040	0.026	10.21 (4)	0.037
2. Model with auto-regressive paths to be constraint to be equal										
a. ASD paths	1.83	2	0.402	1.00	1.00	0.000	0.000–0.037	0.010	0.12 (1)	0.729
b. Prosocial paths	8.76	2	0.013	0.998	0.986	0.035	0.014–0.060	0.024	6.02 (1)	0.014
3. Model with cross-lagged paths constrained to be equal over time										
a. ASD → prosocial path constrained	9.62	2	0.008	0.998	0.984	0.037	0.016–0.062	0.015	7.01 (1)	0.008
b. Prosocial → ASD constrained	4.87	2	0.088	0.999	0.994	0.023	0.000–0.049	0.013	2.08 (1)	0.149
Step 2: Select optimal model										
ii. Reciprocal model with constrained auto-regressive ASD paths, unconstrained auto-regressive prosocial skills paths, unconstrained cross-lagged paths from ASD symptoms to prosocial skills and constrained cross-lagged paths from prosocial skills to ASD symptoms	3.48	3	0.322	1.00	0.999	0.008	0.000–0.034	0.013		

*CFI* comparative fit index, *TLI* Tucker-Lewis Fit Index, *RSMEA* Root Mean Square Error of Approximation, *90% CI* 90% confidence interval of the RSMEA; ∆ symbolizes difference scores between current model and model #1. Model comparison based on difference scores for AIC, BIC and *χ*^2^ values

^a^Chi-square difference calculated using the Satorra–Bentler Scale Chi-square difference test

### Subgroup analyses

Next, we tested the equality of model fit across gender, cohort, ASD diagnostic status, IQ, and self-reported social well-being among classmates. This was done by fixing stability, within-wave correlations, and cross-lagged path coefficients to be equal across subgroups. Fixing across gender, cohort, ASD diagnostic status, IQ and social well-being among classmates did not significantly impair model fit, suggesting that there are no gender differences [∆χ^2^ (7) = 2.62, *p* = 0.918], cohort differences [∆χ^2^ (7) = 11.20, *p* = 0.130], or differences between individuals with and without a clinical diagnosis of ASD [∆χ^2^ (7) = 7.79, *p* = 0.351], with low versus high IQ [∆χ^2^ (7) = 8.22, *p* = 0.313], and with low versus high social well-being in the classroom [∆χ^2^ (7) = 5.96, *p* = 0.545] in the relationship between prosocial skills and ASD symptoms, see Supplementary Table S1 for subgroup estimates.

Furthermore, we fitted the model separately for ASD-related social and communication and ASD-related repetitive symptoms to examine whether the association between prosocial skills and ASD symptoms depended on type of ASD symptoms. Our results showed that, as expected, the parameter estimates for social and communication symptoms were highly similar to the parameter estimates of the full model based on the CSBQ ASD core aggregate score. The cross-lagged effect from *T*1 stereotypic and repetitive behaviours to *T*2 prosocial skills was not significant (*ß* = 0.057*, p* = 0.231), see Supplementary Figure S1.

### Weighted analysis

We reran the optimal RI-CLPM model including the weighting factor to correct for the overrepresentation of cases with (sub)threshold ASD symptom severity in our sample compared to the general population. The result showed similar parameter estimates for the weighted and unweighted model, although the cross-lagged parameter estimate between *T*1 ASD symptoms and *T*2 prosocial skills was now not significant (estimate = 0.106, *p* = 0.110) instead of significant (estimate = 0.132, *p* = 0.029), see Supplementary Figure S2.

## Discussion

This is the first study to examine the dynamical effects between prosocial skills and ASD symptoms over time at the within-person level during adolescence. This is an important asset given that change processes operate within the individual and not at the between-person level, such as when improvements in a person’s prosocial skills lead to reductions in the person’s ASD symptoms over time (i.e., within-person). To study within-person change, we used the recently developed RI-CLPM [38, 39], a novel model that, contrary to the common CLPM, separates the within-person process from stable between-person differences through the inclusion of a random intercept. Main results were that at the between-person level, prosocial skills and ASD symptoms were strongly negatively correlated. At the within-person level, we found that ASD symptoms did not seem to be subject to change under the influence of within-person variability in prosocial skills at an earlier point in time or vice versa. Except for one small positive cross-lagged effect from ASD symptoms at the first assessment wave to prosocial skills at the subsequent wave, all cross-lagged paths were non-significant. In other words, contrary to our hypothesis, we found no evidence that gains in prosocial skills lead to subsequent reduction of ASD symptoms, nor that reductions in ASD symptoms lead to subsequent enhancement of prosocial skills. Rather, the inverse association between prosocial skills and autistic symptoms is highly stable.

The finding that within-person deviations in higher ASD symptoms at age 11 were positively predictive of within-person deviations in higher prosocial skills at age 13 (or lower age 11 ASD symptoms to lower age 13 prosocial skills) is an unexpected finding that contrasts with our hypothesis. The finding indicates that if one’s ASD score at age 11 is higher than expected based on one’s own expected ASD mean at age 11, chances are higher that one has higher than expected prosocial skills at age 13 compared to one’s own expected prosocial mean at age 13. Vice versa, if one’s ASD score at age 11 is lower than expected based on one’s own expected ASD mean, chances are higher that one has lower than expected prosocial skills at age 13 compared to one’s own expected prosocial mean. A possible explanation for this finding may be that prosocial development in adolescence does not follow a linear trajectory. Although there is a general increase in prosocial behaviour from childhood through adulthood [47], there may be declines during adolescence [48–50]. Increasingly more time is spent interacting with peers and peer relations have been found to crucially influence displays of prosocial behaviour in adolescence [50]. Adolescents feel the desire to belong to a social group and group members’ subsequently shape the desired behaviour [51, 52]. It is during this time in development that adolescents start treating friends differently than other peers in terms of prosocial behaviour. For example, 14 year-olds were more generous towards friends than neutral classmates, whereas 10–12 year-olds did not differentiate between these groups in terms of their displays of generosity [53]. Complex group dynamics among peers may be a potential reason why prosocial behaviour declines around age 13. Individuals differ when increases and declines occur [49]. For example, at age 13, lower social skills and a slower growth rate occurred in individuals with autistic disorder compared to individuals with milder ASD (i.e., PDD-NOS) and non-spectrum individuals [54]. Based on this literature on normative prosocial development and individual differences in the timing thereof, we take the positive cross-lagged correlation to mean that typically developing’ adolescents (i.e., those with low ASD scores at age 11) may demonstrate a somewhat decreased prosocial responding around age 13. Conversely, individuals with elevated ASD symptom levels at age 11 may demonstrate a delayed developmental effect of prosocial skills maturation in which prosocial skills in these individuals are still increasing around age 13. The cross-lagged parameter estimate was highly similar, but lost significance after correction for the overrepresentation of cases with (sub)threshold ASD symptom severity in our sample compared to the general population. This suggest that the estimate is less precise (i.e., higher standard error), most likely because less cases in the upper range of symptom severity resulted in a loss of variance in ASD symptoms. This finding substantiates our decision to oversample cases with (sub)threshold ASD symptom severity in our sample.

Of note, the cross-lagged effect was only observed for social and communication problems and not for stereotypic and repetitive behaviours. The two different ASD behaviour domains have only modest phenotypic and genetic overlap [55]. It makes sense that changes in prosocial skills are preceded by changes in social-communication deficits, whereas the association between stereotypic and repetitive behaviours and prosocial skills is more indirect. An example of an indirect association would be that a child who focusses his attention on repetitive behaviours might miss cues for social development or peers may find the repetitive behaviour and limited interests ‘odd’ resulting in fewer social contacts and limited opportunity to practice social behaviour. Moreover, (pro)social skills are still developing in adolescence, and changes can be captured in our model, whereas restricted and repetitive behaviours already decrease during childhood and less during adolescence [56].

As expected, we found that at the between-person level, prosocial skills and ASD symptoms were strongly negatively correlated. This indicates that adolescents whose parents reported higher levels of ASD symptoms across the three measurement waves tend to have lower prosocial skills across the three waves as rated by teachers. This corroborates with previous case control studies in individuals with ASD compared to typically developing children [6, 7]. Our results showed that this between-level negative association between prosocial skills and ASD was also present in children with less severe symptom levels. It is known that ASD symptoms are also present to varying degrees in the general population [56] and with a common genetic background [58, 59]. Just like individuals diagnosed with ASD, individuals with high levels of ASD symptoms were found less proficient on an emotional prosody recognition task [60], had difficulties in recognizing emotional expressions of anger, disgust, and sadness and needed more intense expressions to do so [61], had a reduced ability of implicit social learning (i.e., picking up on the dispositions that other persons held towards them based on combinations of facial expression and gaze direction cues, without being explicitly prompted to) [62], performed less well on a social task that required rating the appropriateness of a character’s responses [63], and generated and selected less-prosocial responses and courses of actions in a scenario task [8]. The literature so far lacked a developmental focus on how mild ASD symptoms and prosocial behaviour are associated. Our findings suggest that the association between reduced prosocial behaviour and increased ASD symptoms is already present at mild (subthreshold) ASD symptom levels and that this association is independent of diagnostic category. This means that there was no qualitative difference between individuals with clinically diagnosed ASD and individuals with milder, non-clinical ASD symptom levels in the longitudinal association between prosocial skills and ASD symptoms, which support a dimensional view of autistic-like behaviours with clinical autism being at the extreme tail of a continuous distribution [57, 64].

Several strengths of this study should be considered when weighing the results. Strengths of this study were (a) the large sample size, (b) the focus on early to late adolescence, which is a critical period in social development and an important developmental context for learning prosocial skills, (c) the repeated measures of ASD symptoms using a well-validated instrument allowing us to chart ASD symptoms over time, and (d) the use of different informants for prosocial skills and ASD symptoms (teacher-, and parent-rated, respectively) to minimize the risk of rater bias. An important limitation was the relatively large number of missing teacher ratings of prosocial behaviour at wave 3. However, incomplete teacher information could be handled using FIML within Mplus [36, 37], and the subsamples with and without missing teacher-rated prosocial behaviour at wave 3 did not significantly differ from each other on mean ASD symptom levels (see note Table 1), suggesting that missingness is unlikely to have biased our results. It should be noted that different teachers scored prosocial skills at each measurement wave. Using different informants each measurement wave to measure the same construct may be considered a general limitation in longitudinal research; however, it is an undeniable reality due to the organization of primary and secondary schooling in The Netherlands. Given that teachers are the natural informants about adolescents’ classroom (prosocial) behaviour, using teacher reports is a strength of this study. Another limitation is that we were dependent on data from the Psychiatric Case Register-North Netherlands (PCR-NN) for information on ASD diagnosis, which is incomplete (i.e., only specialist mental health care (MHC) is covered, while primary youth MHC services, psychiatrists and psychologists in private practice and commercially based MHC services are not included, and specialist MHC use before 2000 was not included), and will likely have errors (i.e., clinical diagnoses in the register may or may not result from standardized structured diagnostic assessment, while diagnostic practices may differ among different settings and/or change over time). Also not all individuals with ASD have been referred for their problems, and not all use health care [21, 65].

A note of caution in the interpretation of our results, finally, is that, although the findings emphasize strong stability of the association between prosocial skills and ASD symptoms, they pertain to developmental variability in an epidemiological context. We cannot derive from our observational study that actively improving prosocial skills, for example, by training these as well as by increasing the opportunity of individuals with high ASD symptoms to practice learned prosocial behaviours (e.g., by helping individuals with ASD broaden their social network), will have little effect on ASD symptoms further on in development. Only long-term follow-up measurement in intervention studies can answer this question.

Taken together, our study expanded knowledge on the association between prosocial skills and ASD symptoms in adolescence. We replicated the already firmly established between-person association between low prosocial skills and high ASD symptoms and showed that this  association is already present at mild (subthreshold) ASD symptom levels, independent of diagnostic category. Our findings further showed no evidence that within-person gains in prosocial skills lead to subsequent reduction of ASD symptoms, nor that reductions in ASD symptoms lead to subsequent enhancement of prosocial skills. We, therefore, conclude from our findings that the inverse association between autistic symptoms and prosocial skills in adolescence is highly stable.

## Electronic supplementary material

Below is the link to the electronic supplementary material.

Supplementary material 1 (DOCX 92 kb)

Supplementary material 2 (DOCX 52 kb)

Supplementary material 3 (DOCX 16 kb)
