# Supplementary material for: Examining the intertwined development of prosocial skills and ASD symptoms in adolescence
Source: Eur Child Adolesc Psychiatry. 2018 Jan 30;27(8):1033–46. doi: 10.1007/s00787-018-1114-3 (PMC6060879; doi:10.1007/s00787-018-1114-3)
Supplement: Supplementary file 1 — Supplementary material 1 (DOCX 92 kb) [file 787_2018_1114_MOESM1_ESM.docx]

**Examining the intertwined development of prosocial skills and ASD symptoms in adolescence**

Anoek M. Oerlemans, Nanda N.J. Rommelse, Jan K. Buitelaar & Catharina A. Hartman

European Child + Adolescent Psychiatry

Anoek M. Oerlemans, University of Groningen, University Medical Center Groningen, Department of Psychiatry, Interdisciplinary Center Psychopathology and Emotion Regulation (ICPE), Groningen, The Netherlands; Department of Cognitive Neuroscience, Donders Institute for Brain, Cognition and Behaviour, Radboud university medical center, Nijmegen, The Netherlands. Email: [a.m.sluiter-oerlemans@umcg.nl](mailto:a.m.sluiter-oerlemans@umcg.nl)

**SUPPLEMENTARY FIGURE S1.** Longitudinal associations between separate ASD traits and prosocial behaviour in the full sample

**.248*****

**.257*****

**.108***

-.024

ơ ^2^ within persons

ơ ^2^ within persons

ơ ^2^ within persons

ơ ^2^ within persons

ơ ^2^ within persons

ơ ^2^ within persons

**-.140*****

.079

-.062

.**129***

.080

**-.615*****

Classroom pro-social behaviour 1

Classroom pro-social behaviour 2

Classroom pro-social behaviour 3

ơ ^2^ between persons

1

1

1

CSBQ ASD 1

CSBQ ASD 2

CSBQ ASD 3

ơ ^2^ between persons

1

1

1

**Social interaction and communication**

.028

.027

*Note*. Asterisks indicate significance of effects (*** *p* < .001, ** *p* < .01, * *p* <.05).

Model fit: *χ*² (3) =4.54, *p* =.21, CFI =1.00, TLI =.998, RSMEA =.014 (90% CI =.000-.037), and SMRS =.012

**.192*****

**.187*****

**.103***

.-.034

ơ ^2^ within persons

ơ ^2^ within persons

ơ ^2^ within persons

ơ ^2^ within persons

ơ ^2^ within persons

ơ ^2^ within persons

**-.094***

.030

-.064

.057

-.053

**-.452*****

Classroom pro-social behaviour 1

Classroom pro-social behaviour 2

Classroom pro-social behaviour 3

ơ ^2^ between persons

1

1

1

CSBQ stereotypic + fear 1

CSBQ stereotypic + fear 2

CSBQ stereotypic + fear 3

ơ ^2^ between persons

1

1

1

**Stereotypic and repetitive behaviours**

-.011

-.011

*Note*. Asterisks indicate significance of effects (*** *p* < .001, ** *p* < .01, * *p* <.05).

Model fit: *χ*² (3) =6.61, *p* =.09, CFI =.999, TLI =.993, RSMEA =.021 (90% CI =.000-.043), and SMRS =.012
