# Supplementary material for: Examining the intertwined development of prosocial skills and ASD symptoms in adolescence
Source: Eur Child Adolesc Psychiatry. 2018 Jan 30;27(8):1033–46. doi: 10.1007/s00787-018-1114-3 (PMC6060879; doi:10.1007/s00787-018-1114-3)
Supplement: Supplementary file 2 — Supplementary material 2 (DOCX 52 kb) [file 787_2018_1114_MOESM2_ESM.docx]

**Examining the intertwined development of prosocial skills and ASD symptoms in adolescence**

Anoek M. Oerlemans, Nanda N.J. Rommelse, Jan K. Buitelaar & Catharina A. Hartman

European Child + Adolescent Psychiatry

Anoek M. Oerlemans, University of Groningen, University Medical Center Groningen, Department of Psychiatry, Interdisciplinary Center Psychopathology and Emotion Regulation (ICPE), Groningen, The Netherlands; Department of Cognitive Neuroscience, Donders Institute for Brain, Cognition and Behaviour, Radboud university medical center, Nijmegen, The Netherlands. Email: [a.m.sluiter-oerlemans@umcg.nl](mailto:a.m.sluiter-oerlemans@umcg.nl)

**SUPPLEMENTARY FIGURE S2.** Weighted estimated from the optimal Random-Intercepts Cross-Lagged Panel Model (RI-CLPM) of the association between ASD symptoms and prosocial behaviour over time in the full sample

**.189****

**.225***

.046

-.055

ơ ^2^ within persons

ơ ^2^ within persons

ơ ^2^ within persons

ơ ^2^ within persons

ơ ^2^ within persons

ơ ^2^ within persons

**-.180*****

.012

-.065

**.**106

.018

**-.460*****

Classroom pro-social behaviour 1

Classroom pro-social behaviour 2

Classroom pro-social behaviour 3

ơ ^2^ between persons

1

1

1

CSBQ ASD 1

CSBQ ASD 2

CSBQ ASD 3

ơ ^2^ between persons

1

1

1

-.003

-.003

*Note*. Asterisks indicate significance of effects (*** *p* < .001, ** *p* < .01, * *p* <.05). The cross-lagged effect between T1 ASD symptoms and T2 prosocial skills became marginally significant after weighting (*p*=.110; unweighted *p*=.029). Model fit: *χ*² (3) =15.60, *p* =.001, CFI =.994, TLI =.972, RSMEA =.039 (90% CI =.021-.059), and SMRS =.020
