# Supplementary material for: Examining the intertwined development of prosocial skills and ASD symptoms in adolescence
Source: Eur Child Adolesc Psychiatry. 2018 Jan 30;27(8):1033–46. doi: 10.1007/s00787-018-1114-3 (PMC6060879; doi:10.1007/s00787-018-1114-3)
Supplement: Supplementary file 3 — Supplementary material 3 (DOCX 16 kb) [file 787_2018_1114_MOESM3_ESM.docx]

**Examining the intertwined development of prosocial skills and ASD symptoms in adolescence**

Anoek M. Oerlemans, Nanda N.J. Rommelse, Jan K. Buitelaar & Catharina A. Hartman

European Child + Adolescent Psychiatry

Anoek M. Oerlemans, University of Groningen, University Medical Center Groningen, Department of Psychiatry, Interdisciplinary Center Psychopathology and Emotion Regulation (ICPE), Groningen, The Netherlands; Department of Cognitive Neuroscience, Donders Institute for Brain, Cognition and Behaviour, Radboud university medical center, Nijmegen, The Netherlands. Email: [a.m.sluiter-oerlemans@umcg.nl](mailto:a.m.sluiter-oerlemans@umcg.nl)

| **SUPPLEMENTARY TABLE S1.** Random intercept cross-lagged panel model estimates for the subgroup analysis | | | | | | | | | | | |
| --- | --- | --- | --- | --- | --- | --- | --- | --- | --- | --- | --- |
|  |  | Gender | | Cohort | | Diagnostic status | | Intelligence | | Social wellbeing among classmates | |
|  |  | Males | Females | Population | Clinical-referred | ASD cases | Non-cases | Low IQ | High IQ | Poor | Good |
|  |  | B | B | B | B | B | B | B | B | B | B |
| Between-person | |  |  |  |  |  |  |  |  |  |  |
|  | Correlation | **-.468***** | **-.667***** | **-.416***** | **-.463***** | **-.276*** | **-.493***** | **-.395***** | **-.674***** | **-.575***** | **-.480***** |
|  |  |  |  |  |  |  |  |  |  |  |  |
| Within-person | |  |  |  |  |  |  |  |  |  |  |
| Correlation | |  |  |  |  |  |  |  |  |  |  |
|  | T1 | **-.189***** | **-.187***** | **-.192***** | **-.100***** | **-.108***** | **-.185***** | **-.152***** | **-.177***** | **-.166***** | **-.199***** |
| Cross-lagged effects | |  |  |  |  |  |  |  |  |  |  |
|  | T1 PS → T2 ASD | -.023 | -.027 | -.040 | -.030 | -.019 | -.028 | -.006 | -.007 | -.002 | -.003 |
|  | T1 ASD →T2 PS | **.134*** | **.105*** | .041 | .063 | .116 | .073 | **.136*** | **.122*** | **.147*** | **.115*** |
|  | T2 PS → T3 ASD | -.021 | -.026 | .029 | -.027 | -.018 | -.028 | -.006 | -.007 | -.002 | -.003 |
|  | T2 ASD → T3 PS | -.023 | -.022 | -.041 | .035 | .026 | .017 | -.007 | -.006 | -.001 | -.001 |
| Stability paths | |  |  |  |  |  |  |  |  |  |  |
|  | T1 PS → T2 PS | **.119*** | **.109*** | **.140**** | **.135**** | **.130**** | **.136**** | .088 | .096 | .108 | .102 |
|  | T2 PS → T3 PS | .001 | .001 | .003 | .003 | .001 | .001 | -.019 | -.018 | -.019 | -.019 |
|  | T1 ASD → T2 ASD | **.331***** | **.324***** | **.231***** | **.282***** | **.321***** | **.286***** | **.347***** | **.328***** | **.293***** | **.272***** |
|  | T2 ASD → T3 ASD | **.386***** | **.351***** | **.284***** | **.237**** | **.296***** | **.322***** | **.363***** | **.392***** | **.302***** | **.311***** |
| Correlated change | |  |  |  |  |  |  |  |  |  |  |
|  | T2 | -.005 | .029 | -.023 | -.015 | .009 | .015 | .019 | .020 | .051 | .060 |
|  | T3 | -.092 | -.010 | **-.114*** | **-.064*** | -.046 | -.083 | -.057 | -.061 | -.059 | -.079 |
| Note. *** *p* <.001, ** *p* <.01, * *p* <.05 | | | | | | | | | | | |
